# Lack or Inhibition of Dopaminergic Stimulation Induces a Development Increase of Striatal Tyrosine Hydroxylase-Positive Interneurons

**DOI:** 10.1371/journal.pone.0044025

**Published:** 2012-09-18

**Authors:** Carla Letizia Busceti, Domenico Bucci, Gemma Molinaro, Paola Di Pietro, Luca Zangrandi, Roberto Gradini, Rosario Moratalla, Giuseppe Battaglia, Valeria Bruno, Ferdinando Nicoletti, Francesco Fornai

**Affiliations:** 1 IRCCS Neuromed, Pozzilli, Italy; 2 Department of Experimental Medicine, University “Sapienza”, Roma, Italy; 3 Department of Functional and Systems Neurobiology, Istituto Cajal CSIC, Madrid, Spain; 4 Department of Physiology and Pharmacology, University “Sapienza”, Roma, Italy; 5 Department of Human Morphology and Applied Biology, University of Pisa, Pisa, Italy; University of Chicago, United States of America

## Abstract

We examined the role of endogenous dopamine (DA) in regulating the number of intrinsic tyrosine hydroxylase-positive (TH^+^) striatal neurons using mice at postnatal day (PND) 4 to 8, a period that corresponds to the developmental peak in the number of these neurons. We adopted the strategy of depleting endogenous DA by a 2-day treatment with α-methyl-*p*-tyrosine (αMpT, 150 mg/kg, i.p.). This treatment markedly increased the number of striatal TH^+^ neurons, assessed by stereological counting, and the increase was highly correlated to the extent of DA loss. Interestingly, TH^+^ neurons were found closer to the clusters of DA fibers after DA depletion, indicating that the concentration gradient of extracellular DA critically regulates the distribution of striatal TH^+^ neurons. A single i.p. injection of the D1 receptor antagonist, SCH23390 (0.1 mg/kg), the D2/D3 receptor antagonist, raclopride (0.1 mg/kg), or the D4 receptor antagonist, L-745,870 (5 mg/kg) in mice at PND4 also increased the number of TH^+^ neurons after 4 days. Treatment with the D1-like receptor agonist SKF38393 (10 mg/kg) or with the D2-like receptor agonist, quinpirole (1 mg/kg) did not change the number of TH^+^ neurons. At least the effects of SCH23390 were prevented by a combined treatment with SKF38393. Immunohistochemical analysis indicated that striatal TH^+^ neurons expressed D2 and D4 receptors, but not D1 receptors. Moreover, treatment with the α4β2 receptor antagonist dihydro-β-erythroidine (DHβE) (3.2 mg/kg) also increased the number of TH^+^ neurons. The evidence that DHβE mimicked the action of SCH23390 in increasing the number of TH^+^ neurons supports the hypothesis that activation of D1 receptors controls the number of striatal TH^+^ neurons by enhancing the release of acetylcholine. These data demonstrate for the first time that endogenous DA negatively regulates the number of striatal TH^+^ neurons by direct and indirect mechanisms mediated by multiple DA receptor subtypes.

## Introduction

Tyrosine hydroxylase (TH)-expressing medium sized aspiny neurons are present in the adult striatum of rodents, monkeys, and humans [1]–[8]. These neurons stain for the high affinity dopamine (DA) transporter [6], [9], and for the GABA-synthesizing enzyme, glutamate decarboxylase (GAD) [3], [9]. In addition, intrinsic TH^+^-neurons of the human striatum express *Nurr1*, a putative specification factor of mesencephalic DAergic neurons [5]. The number of TH^+^-neurons in the adult neostriatum varies considerably in different species, being extremely low in rats and mice (only 10–15 cells in the entire striatum) and high in monkeys (between tens to hundreds of thousands) [1], [2], [6]. What makes these cells potentially relevant to human pathology is their reactivity to DAergic denervation. Chemical lesions of the nigro-striatal DAergic pathway increase the number of striatal TH^+^-neurons in rodents and monkeys [2], [3], [6], [9], [10]. In addition, an increased density of TH^+^ neurons in autoptic striatal samples from patients with Parkinson's disease (PD) has been reported by Porritt et al. [11], but not by Huot et al. [12]. In the latter study, however, all patients had been treated with the DA precursor, L-3′,5′-dihydroxyphenylalanine (L-DOPA) [12]. Remarkably, the number of TH^+^-neurons was reduced in the striatum of individuals affected by Huntington's chorea [12], in which DA concentrations are elevated [13], [14]. These findings suggest that DAergic innervation produces a negative signal that restrains the number of intrinsic striatal TH^+^-neurons [8]. Whether this signal corresponds to DA itself or to other factors that affect cell differentiation or survival is unknown at present.

We have found [15] that the number of intrinsic striatal TH^+^ neurons is elevated in mice during early postnatal life with a peak of 6,000–8,000 cells/hemistriatum at postnatal day (PND) 8, when afferent DAergic axons are scarce and heterogeneously distributed as compared to adult striatum. These DAergic axons are observed as *“clusters”* of DA fibers scattered in the striatum, which produce dense aggregates, defined as “DA islands” [16], [17].

At this age, striatal TH^+^ neurons are found at a relatively long distance (about 50 µm) from clusters of DAergic fibers [15]. The number of TH^+^ neurons sharply decreases at PND16 along with the increase in DAergic innervation [15]. We used PND4-PND8 mice as a model to examine the role of endogenous DA in the regulation of striatal TH^+^ neurons. We adopted the strategy of depleting endogenous DA without affecting the anatomical integrity of the nigro-striatal DAergic pathway, or, alternatively, blocking the action of endogenous DA with the use of subtype-selective DA receptor antagonists.

## Results

### Increased number of striatal TH^+^ neurons following dopamine depletion

**Figure 1:**
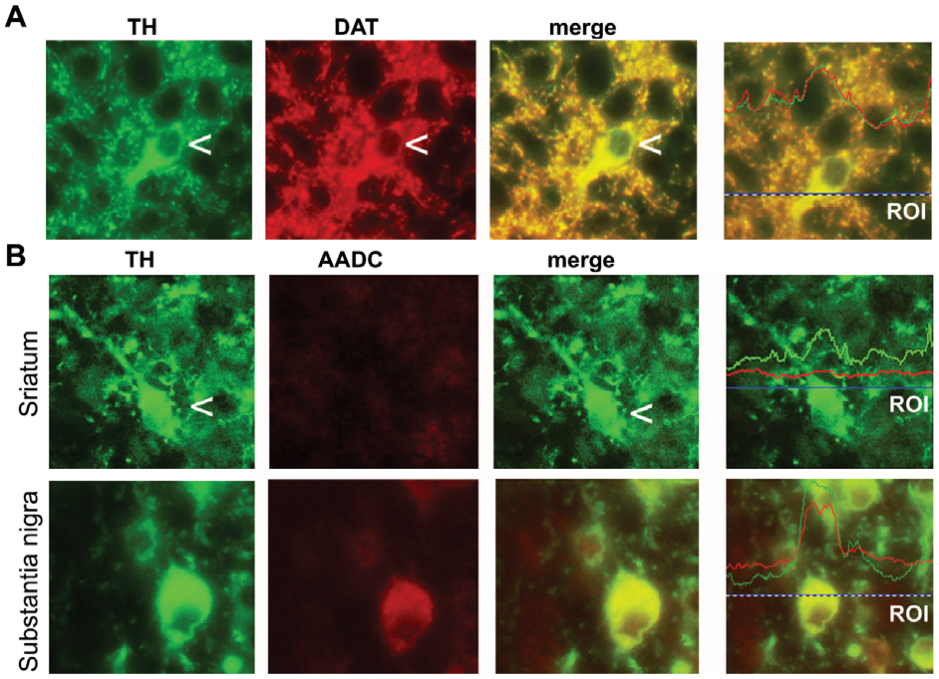
Phenotypic characterization of intrinsic TH^+^ neurons. Double fluorescence staining for TH and DAT, or AADC and for TH and BrdU are shown in (A) and in (B), respectively. Co-localization was examined by densitometric analysis of red and green fluorescence in a selected region corresponding to the horizontal line in the right panels. The coincidence of the fluorescence peaks is indicative of a high level of co-localization.

TH^+^ neurons in the mouse striatum were identified by immunohistochemistry as rounded medium-sized aspiny neurons with a diameter of the cell body of 6±2.3 µm (means+S.E.M; n = 18). These cells account for 3.97±0.21% of the whole striatal NeuN^+^ neuronal population, at PND8. Double fluorescent staining showed that TH^+^ cells stained for the high affinity DA transporter, DAT, which is a selective marker of DAergic neurons, but do not stain for aromatic amino acid decarboxylase (AADC), the enzyme that converts L-3,5,-dihydroxyphenylalanine (L-DOPA) into DA (Fig. 1).

**Figure 2:**
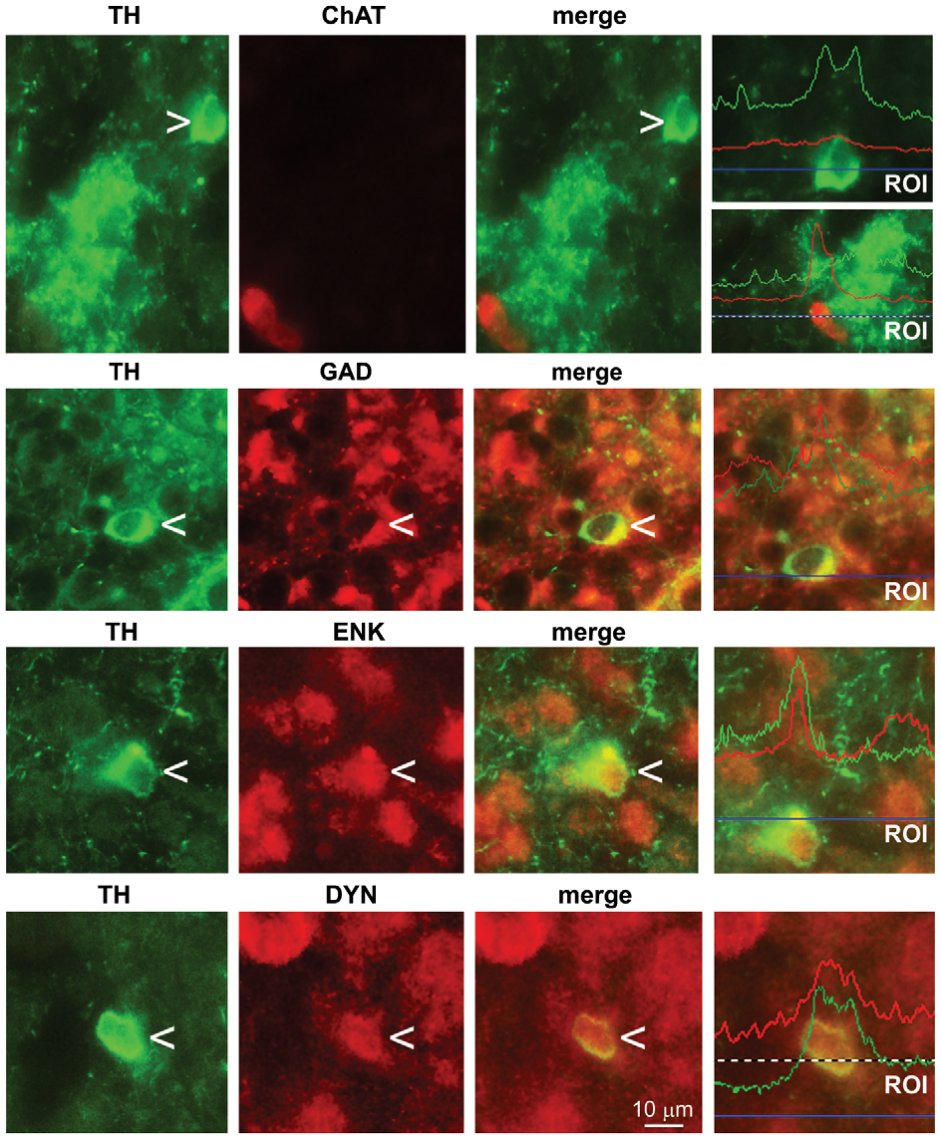
Double fluorescence staining for TH and ChAT, GAD, ENK or DYN. Co-localization was examined by densitometric analysis of red and green fluorescence in a selected region corresponding to the horizontal line in the right panels. The coincidence of the fluorescence peaks is indicative of a high level of co-localization.

We carried out double fluorescent immunohistochemistry to determine whether TH colocalized with GAD (a marker of GABAergic neurons), dynorphin (a marker of striatal projection neurons of the “direct pathway”), enkephalin (a marker of striatal projection neurons of the “indirect pathway”), or choline acetyltransferase (ChAT) (a marker of cholinergic interneurons). TH^+^ cells were immunoreactive for GAD, dynorphin and enkephalins, but nor for ChAT (Fig. 2).

**Figure 3:**
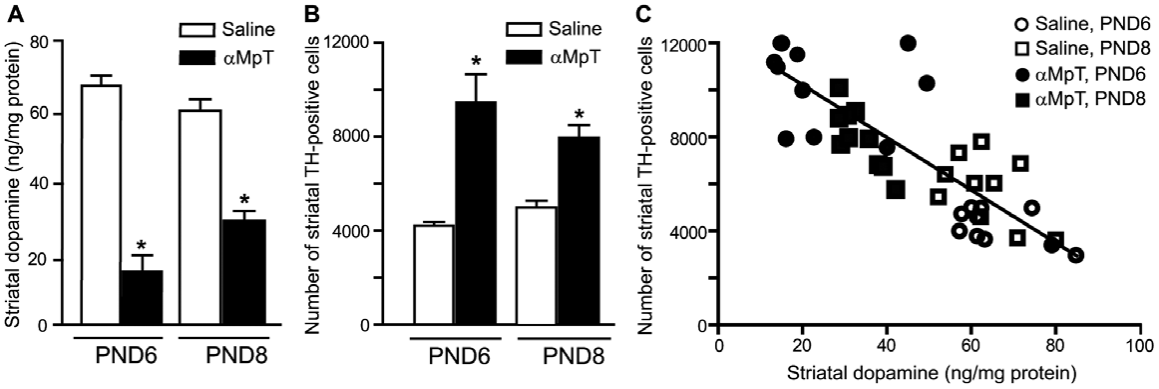
DA depletion increases the number of intrinsic TH^+^ neurons. DA levels and the number of TH^+^ neurons in the striatum of mice treated with αMpT (150 mg/kg, i.p.; injected twice with 24 h of interval at PND4 and PND5), and killed 24 h (PND6) or 72 h (PND8) later are shown in (D) and (E). Values are means+S.E.M. of 10 mice for group. *p<0.05 (Student's test) *vs.* saline-treated mice. Correlation analysis between DA levels and the number of TH^+^ neurons in shown in (F) (r^2^ = 0.65; p<0.05). Empty circles = mice treated with saline and killed at PND6; filled circles = mice treated with αMpT and killed at PND6; empty squares = mice treated with saline and killed at PND8; filled squares = mice treated with αMpT and killed at PND8.

Stereological counting confirmed the developmental peak in the number of striatal TH^+^-neurons at PND8 (total number of TH^+^ neurons per hemistriatum: 1,534±321 at PND1; 3,577±199 at PND4; 4,789±406 at PND6; 6,016±701 at PND8; 1,711±296 at PND14; means ± S.E.M.; n = 6). PND4 mice were treated with the specific TH inhibitor, αMpT (150 mg/kg, i.p., injected twice with 24 h of interval). Mice were killed at PND6 or PND8 (i.e. 24 or 72 h after the last αMpT injection) for measurements of striatal DA levels in left hemistriatum and cell counting in the right hemistriatum. This allowed a correlation analysis between DA levels and the number of TH^+^ neurons. Treatment with αMpT led to a 71.6% reduction in striatal DA levels after 24 h (PND6), followed by a partial recovery (47.5% reduction in DA levels) at 72 h (PND8), as compared to control mice treated with saline (Fig. 3A). Stereological cell counting showed an increased number of striatal TH^+^ neurons in αMpT-treated mice. Cell number increased by two fold at 24 h, and by about 38% at 72 h after αMpT injection (Fig. 3B). We found a high correlation between DA loss and the number of TH^+^ neurons (r^2^ = 0.65; p<0.05) when we pooled all data obtained in mice treated with saline or αMpT and killed at PND6 and PND8 (Fig. 3C).

### Changes in the anatomical distribution of striatal TH^+^ neurons in response to DA depletion

**Figure 4:**
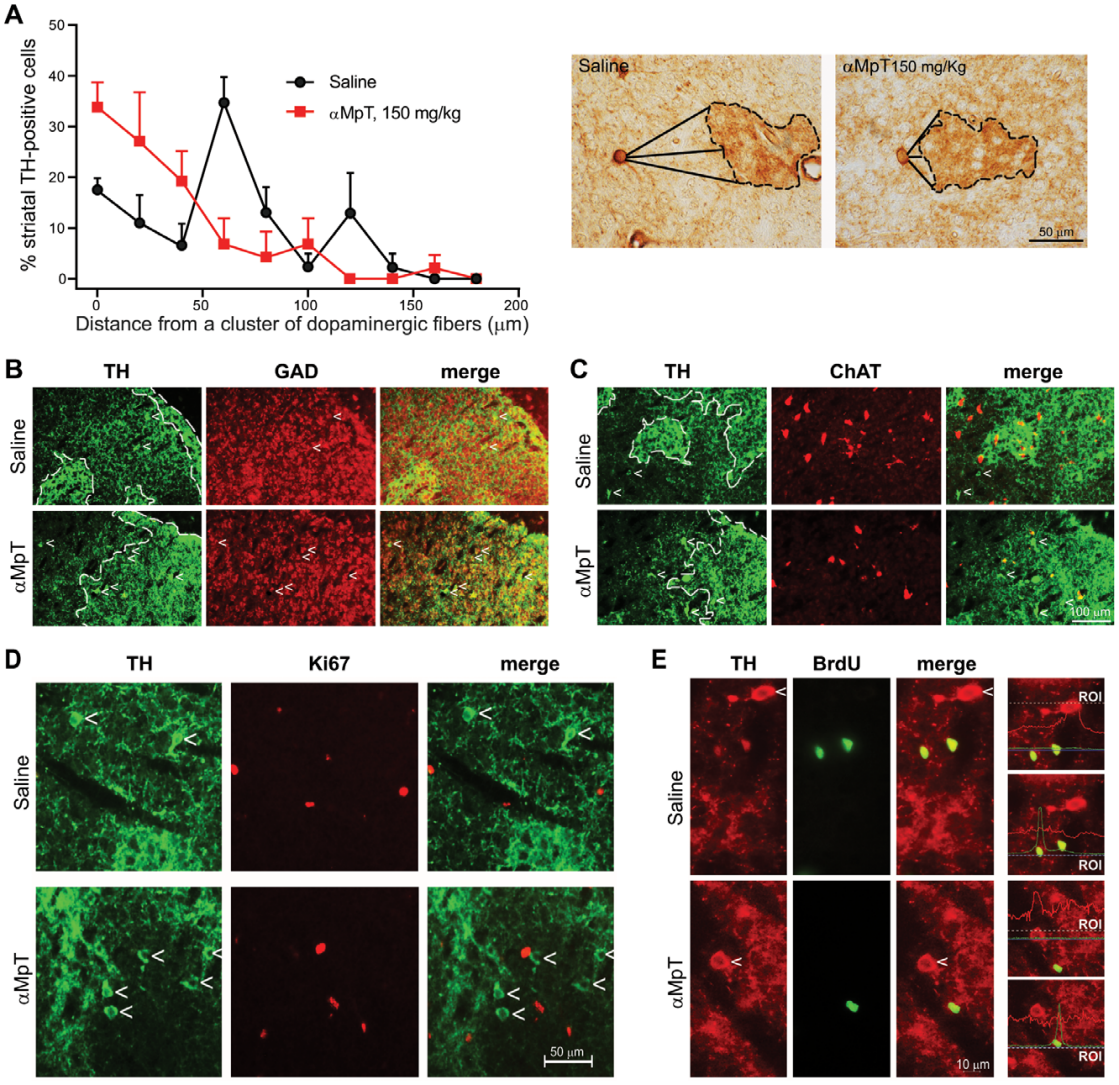
DA depletion changes the spatial distribution of striatal TH^+^ neurons. The distribution profile of TH^+^ neurons in the striatum of mice treated with saline or in striatum of mice treated with saline or αMpT at PND4 (2 injections 24 h apart) and killed at PND6 is shown in (A). Representative images of neurons and fibers stained for TH are shown below the graph. The figure shows the triple vectors used for distance determination. Segments connecting the cell body of TH^+^ neurons to the central border and the two peripheral borders of the clusters are indicated). Note that most of the TH^+^ neurons are placed at shorter distance from the clusters of DA fibers in mice treated with αMpT. Double fluorescence immunostaining for TH and GAD, ChAT, Ki-67, and BrdU in mice treated with saline or αMpT as above is shown in (B), (C), (D), and (E), respectively.

During the first postnatal week striatal striosomes are identified by TH-immunoreactive islands and the surrounding tissue is identified as “matrix” [18]. Dopamine (DA) axons in the developing striatum are scarce and scattered when compared with the adult striatum. During the first postnatal week one can observe dense *“clusters”* of DA axons scattered in the striatum, which produce a patchy image of mesostriatal TH^+^ nerve endings (16,17). Our data showed that treatment with αMpT substantially changes the anatomical distribution of TH^+^ neurons with respect to the cluster of fibers. In control mice treated with saline at PND4 and killed at PND6, most TH^+^ neurons were found at a distance of 60 µm from clusters of TH^+^ fibers, calculated as the average of three segments connecting the cell body of TH^+^ neurons to the central portion and the peripheral borders of the clusters, respectively (Fig. 4A). This distribution pattern is similar to that already seen in untreated mice at PND8 [15] and reveals that the localization of TH^+^ neurons is at the level of the matrix. Mice treated twice with αMpT and killed at PND6 showed clusters of DAergic fibers (“DA islands”) similarly to control mice. However, the distribution of TH^+^ neurons changed dramatically in these mice, with the majority of cells being detected in the close proximity of DAergic fibers (Fig. 4A). Remarkably, 33.83±4.89% of TH^+^ neurons were found inside the clusters in mice treated with αMpT vs. 17.36±2.51% only in mice treated saline (see values corresponding to “0” in the x-axis of Fig. 4A). We wish to highlight that the real number of TH^+^ neurons found at relatively long distance from DA clusters (20–60 µm) did not differ substantially between mice treated with saline and αMpT (3,195±261 and 3,610±184, respectively; n = 10), suggesting that the increased number of TH^+^ neurons in the close proximity of DAergic fibers fully accounts for the difference between saline and αMpT. All TH^+^ neurons stained for GAD, but not ChAT, in both controls and αMpT-treated mice (Fig. 4B,C). In addition, TH^+^ cells found in the close proximity of DAergic fibers in αMpT-treated mice did not colocalize with Ki67 and did not incorporate BrdU, suggesting that these cells are postmitotic and did not derive from an increased proliferation of local neuroprogenitors (Fig. 4D,E).

### Systemic treatment with DA receptor antagonists increased the number of striatal TH^+^ neurons

**Figure 5:**
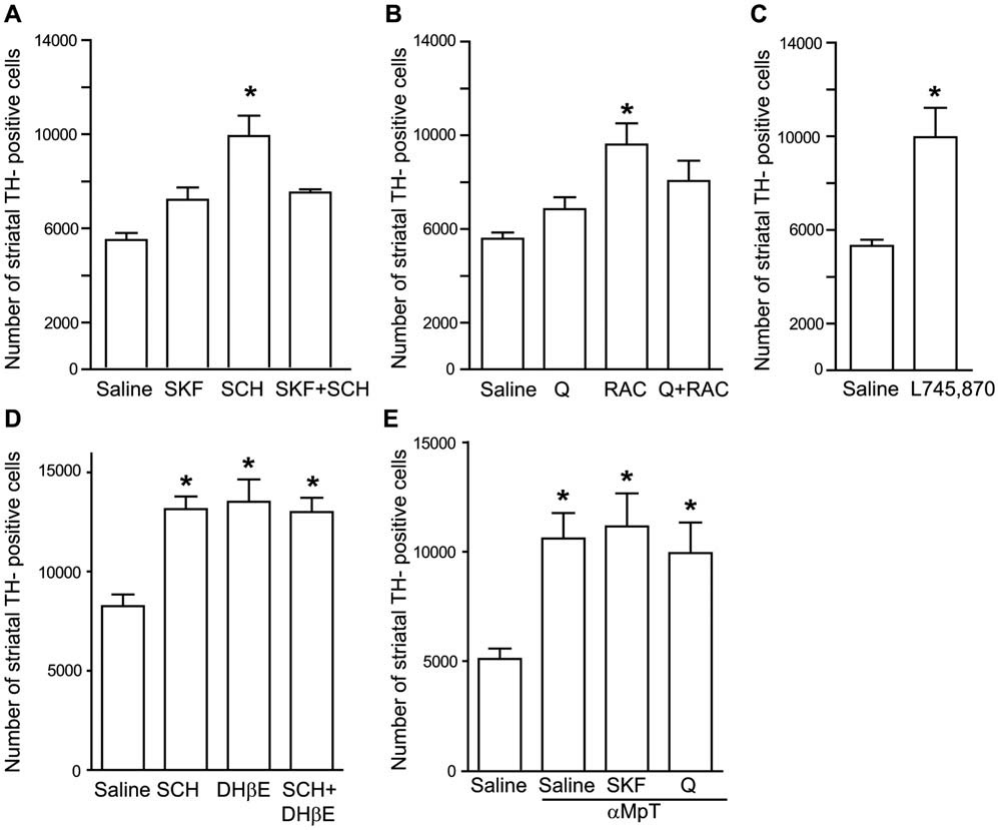
DA and receptors blockade increases the number of striatal TH^+^ neurons. Mice received a single i.p. injection with DA receptor ligands or with a selective nicotinic acetylcholine α4β2 receptor antagonist dihydro-β-erythroidine (DHβE) at PND4 and were killed at PND8. SKF = SKF38393 (10 mg/kg); SCH = SCH23390 (0.1 mg/kg); Q = quinpirole (0.1 mg/kg); RAC = raclopride (1 mg/kg); DHβE = dihydro-β-erythroidine (3.2 mg/kg). (E) PND4 mice subjected to striatal DA depletion by treatment with the TH inhibitor αMpT (150 mg/kg, i.p., twice, with 24 h of interval) were treated with quinpirole (0.1 mg/kg); (n = 6) or SKF38393 (10 mg/kg); (n = 6). Values are means+S.E.M. of 12 (A,C), 17 (B) or 6 (D,E) mice for group. In (A), *p<0.05 (One-way ANOVA+Bonferroni's test) *vs.* all other values; in (B), *p<0.05 (One-way ANOVA+Bonferroni's test) *vs.* values obtained in mice treated with saline or quinpirole alone; in (C,D,E), *p<0.05 (Student's t test) *vs.* values obtained in mice treated with saline.

PND4 mice received a single i.p. injection with the following DA receptor ligands: the D1 receptor antagonist, SCH23390 (0.1 mg/kg); the D1 receptor agonist, SKF38393 (10 mg/kg); the mixed D2/D3 receptor antagonist, raclopride (0.1 mg/kg); the D2-like receptor agonist, quinpirole (1 mg/kg); or the selective D4 receptor antagonist, L-745,870 (5 mg/kg). SCH23390 and raclopride were also injected in combination with SKF38393 and quinpirole, respectively. Mice were killed 4 days later, at PND8. All antagonists injected alone significantly increased the number of TH^+^ neurons in the striatum. The number of TH^+^ neurons increased by 81.4% with SCH23390 (F = 11.41; One-way ANOVA+Bonferroni's test, p<0.05; n = 12); 72% with raclopride (F = 6.21; p<0.05; n = 17) or 120% with L-745,870 (p<0.05; Student's t test, n = 12) (Fig. 5A,B,C). Additional groups of PND4 mice (n = 6) received a single i.p. injection of saline, SCH23390 (0.1 mg/kg), the α4β2 receptor antagonist dihydro-β-erythroidine (DHβE) (3.2 mg/kg) or SCH23390 *plus* DHβE. The number of TH^+^ neurons increased by 56.24% with SCH23390, by 63.86% with DHβE, and by 57.58% with SCH23390 *plus* DHβE (F = 9.886; One-way ANOVA+Bonferroni's test, p<0.05; n = 6) (Fig. 5D). Treatment with SKF38393 or quinpirole did not change the number of TH^+^ neurons either when injected in saline-treated mice either when injected in mice subjected to striatal DA depletion by αMpT treatment (Fig. 5A,B,E). In the groups of mice treated with D1 receptor ligands, values obtained with SCH23390 alone were significantly different from values obtained with SKF38393 alone or with SKF38393+SCH23390 (p<0.05). The number of TH^+^ neurons did not differ among the groups treated with saline, SKF38393 alone, or SKF38393+SCH23390 (Fig. 5A). In the groups of mice treated with D2 receptor ligands, values obtained with raclopride alone were significantly different from values obtained with quinpirole alone (p<0.05), but not with values obtained with raclopride+quinpirole (although raclopride alone increased the number of TH^+^ neurons by 72% *vs.* saline, and raclopride+quinpirole increased the number of TH^+^ neurons by 45% *vs.* saline and 18% *vs.* quinpirole alone). The number of TH^+^ neurons did not differ among the groups treated with saline, quinpirole alone, or quinpirole+raclopride (Fig. 5B).

### Immunohistochemical analysis of DA receptors in the striatum

**Figure 6:**
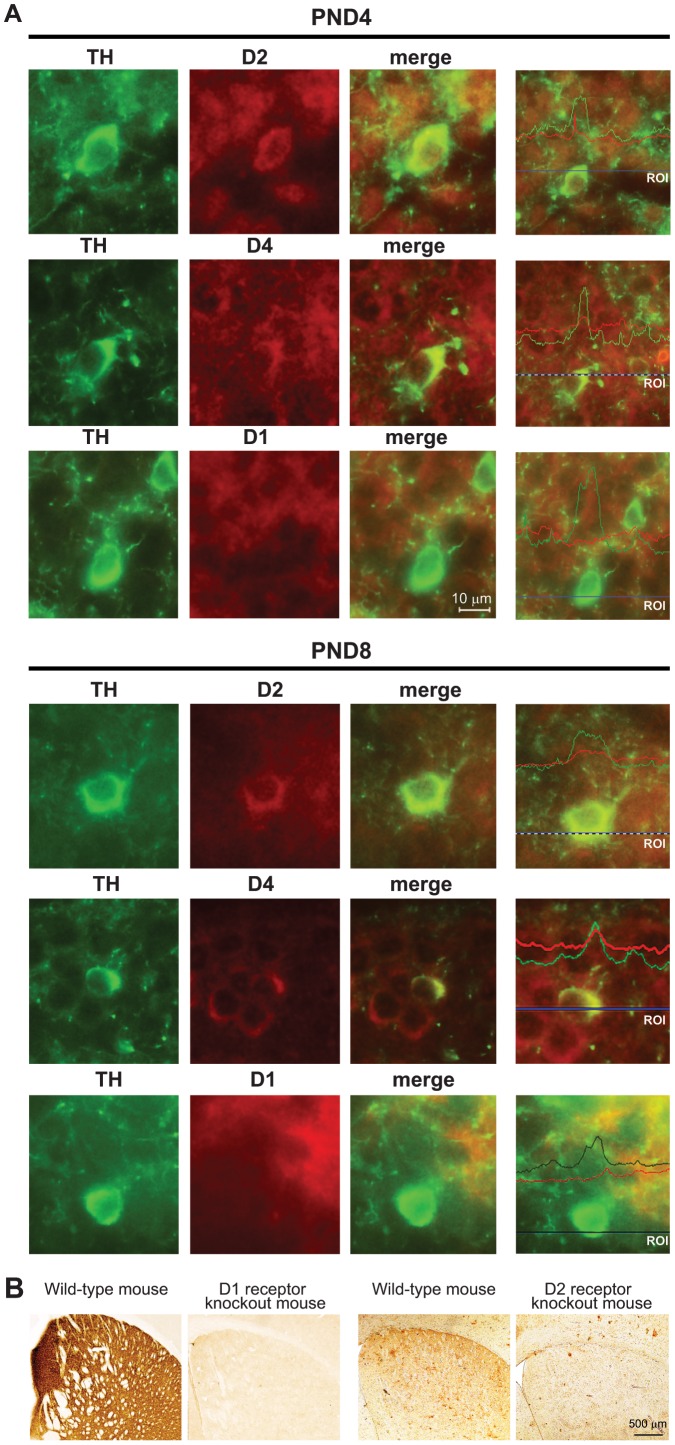
Striatal TH^+^ neurons express D2 and D4 receptors. (A) Double fluorescence staining for TH and D1, D2 or D4 receptors in the striatum of mice at PND4 and PND8 is shown. Co-localization was examined by densitometric analysis of red and green fluorescence in a selected region corresponding to the horizontal line in the right panels. The coincidence of the fluorescence peaks is indicative of a high level of co-localization. (B) Immunoreactivity for D1 and D2 dopamine receptors in striatal sections of adult wild-type and D1 or D2 receptor knockout mice, respectively.

The localization of DA receptor subtypes in striatal TH^+^ neurons was examined in the striatum of PND4 and PND8 mice by double fluorescent staining. At both ages, TH^+^ neurons stained for D2 and D4 receptors. In contrast, D1 receptors were never found in TH^+^ neurons (Fig. 6A).

No immunoreactivity for D1 and D2 dopamine receptors was found in striatal sections of D1 and D2 receptor knockout mice, respectively, which indicates a high specificity of immunostaining (Fig. 6B).

## Discussion

Chemical lesions with 1-methyl-4-phenyl-1,2,3,6-tetrahydropyridine or 6-hydroxydopamine increase the number of striatal TH^+^ neurons in rodents and primates, suggesting that either DA or other factors released from nigro-striatal dopaminergic fibers restrain the number of intrinsic TH^+^ neurons in the striatum [8]. We decided to examine the role of endogenous DA in controlling the number of intrinsic TH^+^ neurons using developing mice (for a detailed characterization of the developmental profile of striatal TH^+^ neurons in mice, see [15]). Striatal TH^+^ cells in mice at PND6–8 expressed DAT, which is a specific marker of DAergic neurons, but did not express AADC, the enzyme necessary for the conversion of L-DOPA into DA. Striatal TH^+^ cells in adult mice treated with 6-hydroxydopamine or methamphetamine were also found to be devoid of AADC [10]. Thus, intrinsic striatal TH^+^ cells in both developing and adult mice may lack the ability to synthesize DA, but they are a potential source for L-DOPA that can be converted into DA by neighbor cells. Intrinsic striatal TH^+^ cells of developing mice were also immunoreactive for the GABA-synthesizing enzyme, GAD, but not for the acetylcholine-synthesizing enzyme, ChAT.

In the striatum, GAD is normally expressed by different populations of interneurons as well as by medium spiny projection neurons of the “direct” and “indirect” pathways [19]. TH^+^ cells were immunoreactive for enkephalin and dynorphins, which are peptide markers for projection neurons of the indirect and direct pathway, respectively [20]. TH^+^ cells apparently expressed D2 receptors (which are normally expressed by projection neurons of the indirect pathway), but not D1 receptors (which are normally expressed by projection neurons of the direct pathway). This particular profile is in agreement with the suggestion that TH^+^ neurons in the developing mouse striatum closely resemble medium spiny projection neurons, but constitute a cell type distinct from classical medium spiny neurons [21].

To elucidate the role of DA in this mechanism, we adopted the strategy of leaving the innervation intact, and depleting endogenous DA with αMpT. We treated mice with αMpT at PND4 and PND5, just prior to the developmental peak in the number of striatal TH^+^ neurons. DA is present in the mouse striatum at birth [22], but no DA release can be detected by microdialysis before PND5 [23]. Thus, we inhibited DA synthesis in a time window that corresponds to the first exposure of the striatal microenvironment to extracellular DA. We found a strong effect of DA depletion on the number of striatal TH^+^ neurons with a highly significant correlation between the extent of DA loss and the increase in TH^+^ neurons. Remarkably, DA loss caused a dramatic change in the distribution of TH^+^ neurons, with most of the newly formed TH^+^ neurons being placed at short distance from DA islands. Our data suggest that DA negatively regulates the number of TH^+^ neurons, and that the distribution of TH^+^ neurons is determined by the concentration gradient of extracellular DA in the developing striatum. It can be argued that in response to DA depletion the majority of TH^+^ cells should still be localized far from DA islands, i.e. at a “safety distance” from the DA that is still produced by, and released from, DAergic fibers. It is possible that the differentiation and spatial distribution of TH^+^ cells is regulated by trophic/attractive signals produced by DAergic fibers and, at the very opposite, by the inhibitory action of DA, which restrains the number of TH^+^ cells in the vicinity of DAergic fibers. Perhaps, when DA levels are reduced in response to αMpT, the unopposed action of these hypothetical trophic signals will substantially increase the number of TH^+^ cells in the vicinity of DAergic fibers, whereas in normal mice they can only support differentiation of TH^+^ cells if concentrations of endogenous DA fall below a critical threshold, i.e. far from the DA islands. This hypothesis is line with two observations: (i) TH^+^ cells are barely detectable before PND4, when the number of DA fibers afferent to the striatum is low [15]; and (ii) in the adult striatum the number of TH^+^ cells increases in response to partial DAergic denervation, whereas TH^+^ cells are no longer detectable in response to a total DAergic denervation [11].

Pharmacological experiments suggested that DA lowers the number of striatal TH^+^ neurons acting at multiple DA receptor subtypes. Drugs that block D1, D2/D3, or D4 receptors all increased the number of TH^+^ neurons, thus mimicking the effects of DA loss. Interestingly, TH^+^ neurons expressed D2 and D4, but not D1 receptors. D2 and D4 receptors are both coupled to Gi proteins [24] and, therefore, a Gi-dependent signaling pathway activated by endogenous DA might restrain TH expression in striatal interneurons. The indirect mechanism whereby endogenous activation of D1 receptors negatively regulates the number of TH^+^ neurons remains to be determined. D1 receptors are localized on striatal cholinergic interneurons, where they facilitate acetylcholine release [25]–[27]. Acetylcholine, in turn, facilitates DA release *via* the activation of presynaptic nicotinic receptors [28]. An interesting possibility is that activation of D1 receptors controls the number of striatal TH^+^ neurons by enhancing the release of acetylcholine, which in turn facilitates DA release from nigro-striatal terminals. The evidence that the nicotinic receptor antagonist, DHβE, mimicked the action of SCH23390 in increasing the number of TH^+^ neurons supports this hypothesis.

The pharmacological specificity of the effects we have seen with DA receptors antagonists was supported by the use SKF38393 and quinpirole, which activate D1-like and D2-like DA receptors, respectively. SKF38393 had no effect on its own and reversed the increase in the number of TH^+^ neurons induced by SCH23390. Mice treated with raclopride+quinpirole showed a trend to a reduction in the number of TH^+^ neurons as compared to mice treated with raclopride alone, although the difference was not statistically significant. The lack of activity of the two agonists alone was unexpected if one assumes that these drugs may diffuse to striatal TH^+^ neurons that are at “safe distance” from endogenous DA. We speculate that activation of DA receptors is necessary, but not sufficient, to negatively regulate the number of striatal TH^+^ neurons. Peptides secreted by nigro-striatal dopaminergic fibers, such as cholecystokinin [29], might have a permissive role in regulating the number of TH^+^ neurons.

The cellular processes that lead to the increased number of striatal TH^+^ cells in response to DA loss is unknown. TH^+^ cells did not express the mitotic marker, Ki-67, and did not incorporate BrdU in both control mice and mice treated with αMpT. In addition, TH^+^ cells of mice treated with αMpT behaved similarly to TH^+^ cells of control mice in expressing the GABAergic marker, GAD. This suggests three potential mechanisms responsible for the increased number of TH^+^ cells in response to DA depletion: (i) the induction of TH in a subpopulation of medium-spiny like GABAergic neurons; (ii) the differentiation of post-mitotic progenitor cells into double TH^+^/GAD^+^ neurons; and or (iii) an increased survival of TH^+^/GAD^+^ neurons that are normally eliminated by the action of extracellular DA between PND4 and PND8. At least in monkeys, new striatal neurons are generated *de novo* throughout the entire lifespan [30], but none of these neurons develop a TH^+^ phenotype even in response to brain-derived neurotrophic factor [31]. Thus, we favor the hypothesis that DA loss or DA receptor blockade de-repress TH expression in a subpopulation of developing GABAergic neurons bearing some of the biochemical features of striatal projection neurons. cAMP enhances TH expression acting at both transcriptional and translational level [32]–[34], suggesting that activation of D2 or D4 receptors by endogenous DA may restrain TH expression by inhibiting cAMP formation. Activation of DA receptors might also affect epigenetic mechanisms that critically regulate TH gene expression [35], [36]. Accordingly, D2 receptor blockade has been shown to rapidly enhance H3 histone acetylation and phosphorylation in the striatum [37].

In conclusion, our data show for the first time that the number of striatal TH^+^ neurons is negatively regulated by endogenous DA acting at multiple DA receptor subtypes. In addition, the demonstration that TH^+^ neurons express D2-like receptors suggests that DA might directly affect the fate of these neurons perhaps regulating TH expression through epigenetic mechanisms. Unraveling these mechanisms might provide new targets for treatments aimed at implementing the number of striatal DAergic cells in PD and other neurodegenerative disorders of the basal ganglia. Whether pharmacological regulation of TH^+^ neurons in the early postnatal life influences the plasticity of the adult striatum in response to nigro-striatal denervation is an interesting question that warrants further investigation.

## Materials and Methods

### Material

α-Methyl-*p*-tyrosine (αMpT) and 5-bromo-2-deoxyuridine (BrdU) were purchased from Sigma (St. Louis, MO). SKF38393, SCH23390, quinpirole, raclopride, L-745,870 and dihydro-β-erythroidine were purchased from Tocris Bioscience (Bristol, UK).

### Ethics Statement

This study (Ricerca Corrente 2010 “Modulation of striatal plasticity” to IRCCS Neuromed Institute) was carried out in strict accordance with the recommendations in the Guide for the Care and Use of Laboratory Animals of the National Italian Institute of Health. The protocol was approved by the Committee on the Ethics of Animal Experiments of the IRCCS Neuromed Institute. Permit Number 432007/A was issued by the Italian Ministry of Health. All efforts were made to minimize suffering. Animals were treated i.p. with drugs and killed by decapitation at different times after treatment.

### Animals

Experiments were performed using CD1 mice (Charles River, Calco, CO, Italy). All mice were kept under environmentally controlled conditions (room temperature = 22°C, humidity = 40%) on a 12-h light/dark cycle with food and water *ad libitum*.

### Experimental design

Striatal DA depletion was induced in PND4 mice by systemic injection of αMpT (150 mg/kg, i.p., twice, with 24 h of interval). Control mice were injected with saline. Mice were killed by decapitation 24 or 72 h after the last injection of αMpT (n = 10) or saline (n = 10) (i.e. at PND6 or PND8). Other groups of PND4 mice (n = 12) received a single i.p. injection of saline or one of the following DA receptor ligands: SKF38393 (10 mg/kg), SCH23390 (0.1 mg/kg), quinpirole (1 mg/kg), raclopride (0.1 mg/kg) or L-745,870 (5 mg/kg), SKF38393 *plus* SCH23390, or quinpirole *plus* raclopride. In order to test the efficacy of DA agonists in a context where D1 or D2 agonists may be competing with endogenous DA, we treated PND4 mice with αMpT *plus* quinpirole (n = 6) or αMpT *plus* SKF38393 (n = 6). In a second experiment, 5 mice per group were treated with saline, quinpirole (1 mg/kg), raclopride (0.1 mg/kg), and quinpirole+raclopride. These data were combined with data obtained in the first experiment, as shown in Fig. 3B). Finally, additional groups of PND4 mice (n = 6) received a single i.p. injection of saline, SCH23390 (0.1 mg/kg), the competitive α4β2 receptor antagonist dihydro-β-erythroidine (DHβE) (3.2 mg/kg) or SCH23390 *plus* DHβE. All mice were killed by decapitation four days after drug injections (i.e. at PND8).

### Bromodeoxyuridine labeling

Mice were systemically injected with saline or αMpT (150 mg/kg, i.p., twice, with 24 h of interval) at PND4 and PND5. The same mice were injected with 3 injections of BrdU (50 mg/kg, i.p., every 2 h) at PND5. Mice were killed 24 h after the last BrdU injection (PND6).

### Monoamine assay

The striatum was homogenized by sonication in 0.6 ml of ice-cold 0.1 M PCA, and DA levels were measured by HPLC with electrochemical detection as described previously [38].

### Immunohistochemical analysis

Brains were dissected out, fixed in ethanol (60%), acetic acid (10%), and chloroform (30%), and included in paraffin. Tissue sections (10 µm) were incubated overnight with monoclonal mouse anti-TH (1∶200; Sigma) or monoclonal mouse anti-NeuN (1∶100; Millipore, Billerica, MA) antibodies and then for 1 h with secondary biotin-coupled anti-mouse antibodies (1∶200; Vector Laboratories, Burlingame, CA). 3,3-Diaminobenzidine tetrachloride (Sigma) was used for detection.

Double fluorescence immunostaining was performed by incubating the sections overnight with monoclonal anti-TH antibodies (mouse; 1∶50; Sigma; code: T1299) and polyclonal antibodies recognizing D1 receptors (rabbit; 1∶20; Santa Cruz, CA; code: sc-14001), D2 receptors (goat; 1∶20; Santa Cruz; code: sc-7522), D4 receptors (rabbit; 1∶20; Chemicon, Tecumela, CA; code: AB1787P), choline acetyltransferase (ChAT) (goat; 1∶100; Millipore; code: AB144P), glutamic acid decarboxylase (GAD) (rabbit; 1∶100; recognizing both GAD_65_ and GAD_67_; Sigma; code: G5163), the high affinity dopamine transporter (DAT) (rat; 1∶50; Millipore; code: MAB369), Aromatic L-amino acid decarboxylase (AADC) (rabbit; 1∶100; Enzo Life Sciences, Farmingdale, New York; code: BML-AZ1030), Leu-Enkephalin (ENK) (rabbit; 1∶50; Millipore; code: AB502), Dynorphin A (rabbit; 1∶100; Abcam; code: ab11134), Ki67 (rabbit; 1∶100; Spring Bioscience Pleasanton, CA; code: M3060) and then for 1 h with secondary fluorescein-conjugated anti-mouse antibodies (1∶100; Vector) and Cy3-conjugated anti-rabbit, anti-goat or anti-rat antibodies (1∶500; Chemicon). Before incubation with primary antibodies, sections were treated with 10 mM, pH 9.0, Tris-EDTA buffer, and heated in a microwave for 10 min for antigen retrieval.

Double fluorescence immunostaining for TH and BrdU was performed by incubating the sections with a polyclonal anti-TH antibody (rabbit; 1∶200; Sigma; code: SAB2103892) and a monoclonal anti-BrdU antibody (mouse; 1∶10; BD Biosciences, San Jose, CA; code: 347580). For an optimal BrdU immunostaining, sections were incubated in 1 N HCl for 60 min at room temperature and then with 0.1 M sodium tetraborate for 10 min. After overnight incubation with anti-BrdU antibody, sections were incubated with secondary fluorescein-conjugated anti-mouse (1∶100; Vector) for 1 h. After an extensive washing, section were incubated overnight with anti-TH antibody and then with secondary Cy3-conjugated anti-rabbit antibodies (1∶100; Vector) for 1 h at room temperature. Before incubation with primary antibodies, sections were incubated in citrate buffer (10 mM, pH 6.0) or in Tris-EDTA buffer (10 mM, pH 9.0) and heated in a microwave for 10 min for BrdU or TH antigen retrieval, respectively.

Co-localization of proteins was examined by densitometric analysis of green and red fluoresce in selected microscopic regions.

The specificity of the antibodies used for immunohistochemical analysis of D1 and D2 dopamine receptors was performed using striatal tissues coming from D1 and D2 knockout mice (Figure 6B).

Tissue sections (10 µm) were incubated overnight with polyclonal antibodies recognizing D1 receptors (rabbit; 1∶20; Santa Cruz, CA) or D2 receptors (goat; 1∶20; Santa Cruz) and then for 1 h with secondary biotin-coupled anti-rabbit (1∶200, Vector Laboratories) or anti-goat antibodies (1∶500; Vector Laboratories). 3,3-Diaminobenzidine tetrachloride (Sigma) was used for detection.

### Cluster analysis

We measured the regional distribution of TH^+^ striatal cells with respect to TH^+^ fiber clusters by tracing a calibrated straight line connecting each TH^+^ striatal cell and the nearest cluster of fibers. We traced three segments connecting the cell body of TH^+^ cells to the central border and to the inferior and superior border of the clusters (see image in Fig. 2A), respectively, and we calculated the mean length of the three segments for each determination.

### Stereological cell counting

The number of TH^+^ cells in the striatum was assessed by stereological technique and an optical fractionator using a Zeiss Axio Imager M1 microscope equipped with a motorized stage and focus control system (Zeta axis), and with a digital video camera. The software Image-Pro Plus 6.2 for Windows (Media Cybernetics, Inc., Bethesda, MD) equipped with a Macro was used for the analysis of digital images. The Macro was obtained by Immagine and Computer, Bareggio, Italy and the characteristics of this Macro are published [39]. The analysis was performed on 6 sections of 30 µm, sampled every 300 µm on the horizontal plan of the striatum, in which the striatum was identified and outlined at 2.5× magnification. TH^+^ or NeuN^+^ cells were counted at 100× magnification as described [40]. For stereological analysis, we used a grid of disectors (counting frame of 100×75 µm; grid size 300×300 µm), with 1.3 as numerical aperture of the lens. The striatum volume, calculated according the Cavalieri method, was 2±0.5 mm^3^ for each striatum as assessed in six sections of 30 µm cut every 300 µm on the horizontal plan of the striatum.

The total number of TH^+^ cells per hemistriatum was computed from the formula: N = Σ(n)×1/SSF×1/ASF×1/TSF, where n is the total number of cells counted on each disector; SSF (fraction of sections sampled) the number of regularly spaced sections used for counts divided by the total number of sections across the striatum ( = 1/6); ASF (area sampling frequency) the disector area divided by the area between disectors (7500 µm^2^×disector number/region area); and TSF (thickness sampling frequency) the disector thickness divided by the section thickness (20 µm/30 µm).
